# Liver transplantation: embracing the future with machine perfusion

**DOI:** 10.1590/0102-672020260000017e1946

**Published:** 2026-07-27

**Authors:** Henrique de Aguiar WIEDERKEHR, Michelle C. NGUYEN, Adyr MOSS, Amit Kumar MATHUR, Rafael NUÑEZ-NATERAS, Julio Cesar WIEDERKEHR, Júlio Cezar Uili COELHO

**Affiliations:** 1 Mayo Clinic, Division of Transplant Surgery - Phoenix, Arizona, USA.; 2 Universidade Federal do Paraná, Department of Surgery - Curitiba (PR), Brazil.

**Keywords:** Liver Transplantation, Transplantation, Organ Transplantation, Perfusion, Tissue and Organ Procurement, Transplante de Fígado, Transplante, Transplante de Órgãos, Perfusão, Obtenção de Tecidos e Órgãos

## Abstract

Despite the annual increase in liver transplants, a persistent disparity between suitable donors and recipients continues to challenge the transplant community. The introduction of machine perfusion emerged as a novel approach to organ preservation. This technique, which utilizes extended criteria donor grafts, is expected to reduce rejection rates and expand the donor pool. Its principle, like that of any *ex vivo* machine perfusion, is based on the continuous supply of nutrients and oxygen to maintain cellular metabolism while interrupting anaerobic processes. This helps prevent conditions such as ischemia-reperfusion injury, early graft dysfunction, and ischemic cholangiopathy, which can lead to graft loss and the need for retransplantation. There are two main modes of *ex vivo* machine perfusion: hypothermic and normothermic, differentiated by the operating temperature. Utilizing *ex situ* perfusion machines presents a promising opportunity to increase liver graft usage and reduce waitlist times. However, it is crucial to evaluate how much this technology would raise the overall cost of transplants. In Brazil, implementing a machine perfusion program may encounter unique challenges not seen in other countries. These challenges include limited access to the healthcare system for high-risk liver transplant candidates, long waiting lists, inadequate support for organ donors, reliance solely on deceased brain-dead donors, since donation after cardiac death is not ethically accepted, and geographical barriers.

## ARTICLE HIGHLIGHTS

• Machine perfusion represents a revolution in organ preservation for liver transplantation.

• With over 67,000 patients on the waiting list in 2024, Brazil urgently needs new organ preservation strategies.

• Machine perfusion has the potential to save hundreds of discarded livers per year, expanding the donor pool.

• The cost of normothermic machine perfusion can be justified by increased graft utilization and reduced retransplantation rates.

## Summary

**CENTRAL MESSAGE:**

Despite the annual increase in liver transplants, the persistent disparity between suitable donors and recipients remains an ongoing issue for the transplant community. Alternatives such as extended criteria donor allografts, donors after circulatory death, and grafts with significant macrosteatosis have been employed to address this disparity. The introduction of machine perfusion in clinical practice emerged as a novel approach to organ preservation. This technique is expected to lower rejection rates and broaden the donor pool by utilizing extended criteria donor grafts.

**PERSPECTIVES:**

The advent of the perfusion era in liver transplantation rapidly modified the landscape for the predictable future. New research and randomized controlled trials are underway to determine whether machine perfusion is a viable and superior alternative to static cold storage. It remains uncertain how costs will affect healthcare expenditures related to transplants, as machine perfusion has shown improved graft utilization and better patient outcomes. Comprehensive and standardized transplant research studies are essential to thoroughly investigate these issues and provide robust data to support the use of machine perfusion.

## VISUAL ABSTRACT

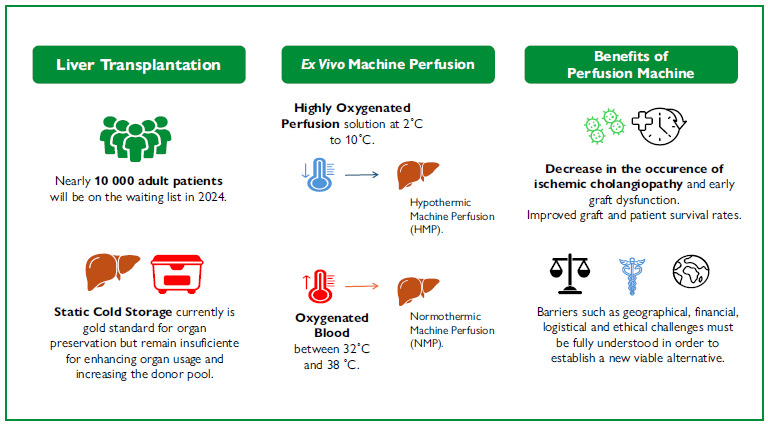

## RESUMO

Apesar do aumento anual no número de transplantes hepáticos, persiste uma disparidade entre doadores adequados e receptores, desafiando a comunidade transplantadora. A máquina de perfusão surge como inovação na preservação de órgãos, especialmente ao utilizar enxertos de doadores com critérios expandidos, podendo reduzir taxas de rejeição e ampliar o *pool* de doadores. Seu princípio, como o de qualquer perfusão *ex vivo*, baseia-se na oferta contínua de nutrientes e oxigênio, mantendo o metabolismo celular e interrompendo processos anaeróbicos. Esse mecanismo ajuda a prevenir lesões por isquemia-reperfusão, disfunção precoce do enxerto e colangiopatia isquêmica, complicações associadas à perda do enxerto e à necessidade de retransplante. Existem duas modalidades principais de perfusão *ex vivo* em máquina: hipotérmica e normotérmica, diferenciadas pela temperatura operacional. A utilização dessas tecnologias representa uma oportunidade promissora para aumentar o aproveitamento de fígados e diminuir o tempo de espera. Contudo, é necessário avaliar até que ponto elas aumentariam os custos dos transplantes. No Brasil, a implantação dessa técnica pode enfrentar desafios específicos não encontrados em outros países. Os desafios incluem o acesso limitado ao sistema de saúde por pacientes em situação de alto risco, listas longas, apoio insuficiente aos doadores, dependência exclusiva de doadores falecidos com morte cerebral, já que a doação após parada cardíaca não é eticamente aceita no país, além de barreiras geográficas relevantes.

## INTRODUCTION

Despite the annual increase in liver transplants, the persistent disparity between suitable donors and recipients remains an ongoing issue for the transplant community^60^. As per Organ Procurement and Transplantation Network data^6^, nearly 10,000 adult patients were on the waiting list in 2024 in the United States. The number of patients on the waiting list in the same year in Brazil was 67,879^6^ ^,^ ^33^.

Alternatives such as extended criteria donor (ECD) allografts, donation after circulatory death (DCD), and grafts with significant macrosteatosis have been employed to address this disparity^22^. The use of grafts from ECD is more susceptible to ischemia-reperfusion injury, early graft dysfunction (EAD), and ischemic cholangiopathy (IC) when preserved under static cold storage (SCS), the current standard method for organ preservation^41^ ^,^ ^57^.

Those constraints were always a subject of interest, and many studies intended to fully understand the mechanisms of this injury and the need to develop new preservation methods.

The introduction of machine perfusion in clinical practice emerged as a novel approach to organ preservation. This technique is expected to lower rejection rates and broaden the donor pool by utilizing ECD grafts.

Current studies are being conducted to determine the advantages of machine perfusion in the context of transplantation.

The beginning of machine perfusion: a revolutionary advancement

Claude Bernard, a French physiologist, was the first to describe an ex vivo liver perfusion model, with ongoing studies on isolating glycogen from the liver and discovering the process of gluconeogenesis in 1850^36^.

In 1935, Lindbergh and Carrel developed the prototype of a machine for organ perfusion. This device consisted of a glass pump that preserved animal organs outside the body by pushing “artificial blood” through the pump and into the organ via a tube connected to the artery of the organ^16^. This innovation ultimately contributed to the development of the heart-lung machine.

The efforts to establish a new preservation method were unsuccessful, and the principles of SCS became the gold standard for organ preservation.

Brettschneider et al. first reported the advent of *ex situ* perfusion machines in 1967 in an animal model, which was later applied to the first series of human liver transplantations by Thomas Earl Starzl^7^. However, the research was discontinued after unsuccessful attempts, especially after the introduction of the University of Wisconsin solution.

Due to the increasing demand for alternatives to reduce waiting lists and expand the donor pool, new studies have been conducted on various perfusion methods, including *ex situ* perfusion machines.

Guarrera et al. performed the first clinical trial with hypothermic machine perfusion (HMP) in 2010^26^. In 2013, Vogel et al. performed the world’s first liver transplant with the normothermic machine perfusion (NMP) in England^45^ ^,^ ^57^.

Those studies were considered landmarks within the transplant community and initiated a series of studies to establish *ex vivo* machine perfusion as a viable option for transplantation.

### Static cold storage current perspectives

SCS, widely recognized as the gold standard for organ preservation, employs a meticulous process that flushes the organs thoroughly with a carefully formulated preservation solution. This solution is stored at optimal temperatures between 2-4°C^10^.

The underlying principle of this method is that the reduced temperature significantly slows down cellular metabolism. This effectively decreases the cells’ energy demands and interrupts anaerobic metabolic processes, which can be detrimental during periods of storage.

A key characteristic of this preservation technique is the specially designed preservation solution itself. This solution contains vital cell-impermeant agents such as lactobionic acid, raffinose, and hydroxyethyl starch. These components work together to create an ideal biological environment, preventing significant cell swelling during the essential time for cold ischemia. Moreover, the solution contains glutathione and adenosine, two fundamental compounds that assist in metabolic restoration once the organs are ready for reperfusion^54^.

Over time, many organ preservation solutions were developed, such as Euro-Collins, the University of Wisconsin, histidine-tryptophan-ketoglutarate, and more recently, the Institut George Lopez^30^.

Despite ongoing efforts and improvements, the SCS remains insufficient for enhancing organ usage and increasing the donor pool.

### Ex vivo machine perfusion

The principle of any *ex vivo* machine perfusion is to offer nutrients and oxygen delivery for cellular metabolism and interrupt anaerobic metabolism to prevent conditions such as ischemia-reperfusion syndrome, EAD, and IC, that can culminate in graft loss and ultimately retransplantation.

**Figure 1.**
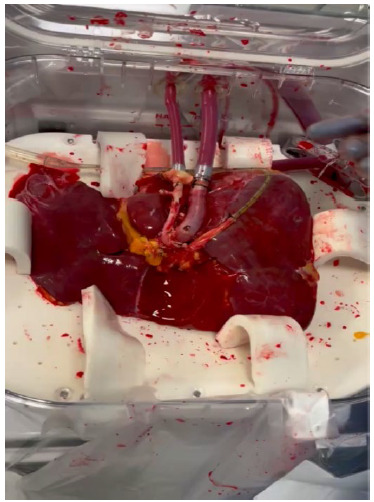
Shows a liver after being placed on the Organ Care System Liver normothermic machine perfusion. Three cannulas have been inserted: one into the portal vein, another into the arterial vasculature, and a third into the common bile duct.

There are two main types of *ex vivo* machine perfusion: HMP and NMP. The key difference between these two methods is the temperature at which they operate. HMP perfuses the organ’s vascular system at temperatures typically ranging from 2-10°C. In contrast, NMP perfuses at a temperature range of 34-37°C, which closely mimics the body’s physiological state (Figure 1).

There is also a new *ex vivo* machine perfusion subtype called subnormothermic machine perfusion (SNMP). It is performed at approximately 20°C with oxygenated perfusate and has shown promise in initial preclinical studies^2^ ^,^ ^3^ ^,^ ^29^ ^,^ ^62^. However, its benefits compared to NMP and HMP are still unclear. Also, those studies included a limited duration of perfusion, and animal studies are not yet applicable to humans. More studies must be conducted to understand the role of SNMP in perfusion strategies.

### Hypothermic machine perfusion

HMP quickly became the focus of most studies. An important modification was the hypothermic oxygenated perfusion (HOPE) that used a highly oxygenated (pO_2_ >60 kPa) artificial solution at hypothermic temperatures, i.e., 8-12°C^53^.

The circulating perfusate is delivered through the portal vein, and dual hypothermic oxygenated perfusion (D-HOPE) devices circulate perfusate through both the portal vein and the hepatic artery. Currently, no clinical studies are available comparing the two different strategies.

Many groups in Europe frequently use HMP devices, but currently, no such devices are approved by the US Food and Drug Administration (FDA) for clinical use, despite ongoing clinical trials^1^ ^,^ ^8^ ^,^ ^43^.

Pereyra et al. recently presented the first analysis of real-world data comparing HOPE modalities with SCS regarding outcomes after liver transplantation. HOPE-treated organs display a reduced incidence of biliary complications and shorter hospitalization^47^.

A meta-analysis conducted by Parente et al. considered four randomized controlled trials (RCTs) regarding the use of HOPE among all RCTs about machine perfusion. All four RCTs compared this perfusion strategy with SCS. A total of 482 patients were analyzed, divided into two groups: 241 patients received a graft after HOPE treatment, and 241 livers were cold stored^14^ ^,^ ^50^ ^,^ ^52^ ^,^ ^55^.

All four randomized HOPE studies reported EAD rates, which occurred at 18.6% in the HOPE group compared to 40.2% in the SCS group, representing a lower risk for EAD. Also, overall biliary complication rates were lower in the HOPE group. However, this analysis did not reach statistical significance; the effect was graded as moderate, with a likely 6.9% reduction in overall biliary complications. HOPE significantly reduced graft loss and retransplantation rates compared to cold storage. Such effects were graded with high and moderate certainty, respectively^21^ ^,^ ^47^.

A recently published multicenter observational cohort study focusing on HOPE/D-HOPE treated liver transplants analyzed 1,202 liver transplant recipients from 22 European centers. The study reported impressive death-censored graft survival rates of 94% at one year, 90% at three years, and 87% at five years. Additionally, the overall patient survival rates were 91% at one year, 86% at three years, and 81% at five years^17^.

### Normothermic machine perfusion

NMP is based on mimicking physiological conditions, since it provides high-pressure pulsatile oxygenated, nutrient-rich blood between 32-38°C through the hepatic artery and low-pressure non-pulsatile flows in the portal vein, preserving metabolic function. Compared to other preservation methods, the primary advantage of NMP is its ability to conduct viability testing prior to transplantation. This testing can evaluate hepatocellular viability by examining the liver’s metabolic functions through real-time analysis of lactate clearance, bile production, and various biochemical components, such as pH, glucose reabsorption, and bicarbonate secretion.

There are currently no universal set criteria for determining the viability of livers after undergoing NMP, although groups from Cambridge and Birmingham have tried to establish relevant viability criteria on which many current protocols are based^35^ ^,^ ^58^.

The VITTAL clinical trial, a prospective, non-randomized, phase 2 trial, conducted by the Birmingham group, performed viability assessment on discarded livers after the use of NMP. Of the discarded livers that were perfused, 71% were transplanted with 100% 90-day patient and graft survival^35^.

**Figure 2.**
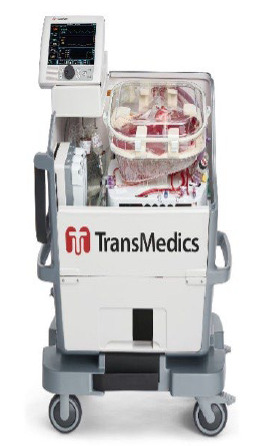
The Organ Care System Liver is an integrated system composed of three main components: the liver console, a perfusion set, and a combination of medications, solutions, and bile salts for infusion (TransMedics^®^). This platform is designed to perfuse both the portal venous and hepatic arterial circulations using warm, oxygenated, and nutrient-enriched blood-based perfusate.

**Figure 3.**
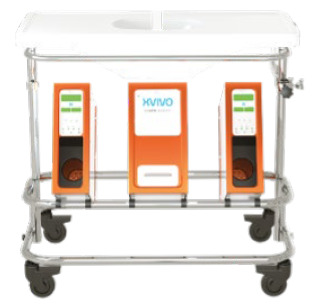
XVIVO’s Liver AssistT^®^ has an integrated organ reservoir, dual circuits for independent pressure-controlled perfusion of both the portal vein and hepatic artery, and can operate at temperatures from below 12°C to 38°C, allowing it to be used for both hypothermic oxygenated perfusion, normothermic machine perfusion, sub-normothermic, or a combination, of these techniques.

The first NMP device was approved clinically in the United States in late 2021, and there are currently two FDA-approved, commercially available NMPs for liver transplantation in the US: the Organ Care System (OCS, TransMedics Inc^®^, Andover, Massachusetts, USA) (Figures 2 and 3) and OrganOx^®^ (Metra, Oxford, UK)^42^ ^,^ ^46^. Only the OCS from TransMedics^®^ is currently being used in the donor hospital, therefore it can be transported to the recipient’s hospital.

OrganOx^®^ is allocated only at the recipient’s hospital, therefore the livers are first procured and placed in SCS and then transported to the transplant hospital where the organ is placed on the pump^46^.

The *ex vivo* machine perfusion is currently being used as an alternative to preserve ECD and DCD grafts. Therefore, the main topic of discussion in the transplant community is the incidence of IC and EAD in pumped livers.

IC is a condition that can lead to readmissions and even retransplantation. It is often associated with DCD grafts, since the longer donor warm ischemia time predisposes to ischemic insult during retrieval^28^ ^,^ ^32^, with previously reported incidences reaching as high as 47%^18^ ^,^ ^32^, with 11.4% at experienced transplant centers^12^.

The PROTECT trial, a US multicenter randomized clinical trial, compared post-transplant outcomes for recipients who received donor livers preserved using SCS or the OCS Liver. A total of 115 cases were divided into two groups: one group with grafts that underwent SCS (61 cases) and the other group with grafts that were perfused by NMP (54 cases). The study showed a significant reduction in the incidence of IC in the OCS-liver group compared with those using SCS (3 *vs*. 11%). Also, the study demonstrated a significant decrease in the incidence of EAD compared with SCS in the primary analysis (18 *vs*. 31%). Therefore, the study concluded that the NMP group revealed superior short-term and mid-term clinical outcomes and a higher number of donor livers used for transplantation^34^.

One of the hypotheses for the reduction of IC in NMP grafts is that the perfusion machine may function, since it uses continuous flow, as a thrombolytic therapy before transplanting the organ. Therefore, the microthrombi in the peribiliary vascular plexus were not able to form non-anastomotic biliary strictures^20^.

IC usually develops within three to six months after transplantation, notwithstanding it can occur up to one year following surgery^25^ ^,^ ^27^. The time of the diagnosis is dependent on the type of imaging modalities used and the frequency of imaging, as determined by periodic surveillance protocols or guided by clinical or laboratory findings^12^.

Biliary strictures are characteristic imaging findings of IC in the setting of DCD liver transplantation, and they can be divided into anastomotic and non-anastomotic strictures (NAS).

NAS consists of areas of biliary injury and narrowing other than the biliary anastomosis and mostly involves the donor intrahepatic ducts proximal to the anastomosis^51^ ^,^ ^56^.

Endoscopic retrograde cholangiopancreatography (ERCP)-guided biliary intervention is currently the cornerstone of therapy for patients who develop IC after liver transplantation, with balloon dilation and placement of plastic stents in all accessible strictures. The goal is to relieve obstruction and prevent recurrent cholangitis.

The ultimate treatment for NAS in the DCD liver transplant setting is the retransplantation with rates of 47.7% even in experienced transplant centers^12^. Although there is still a strong association between NAS and DCD allografts, it seems that NMP can mitigate this incidence when compared to SCS preservation, even in longitudinal follow-up^62^. Recent data have demonstrated that the incidence of IC, EAD, and primary nonfunction in DCD grafts is nearly equivalent to grafts from donation after brain death (DBD)^9^ ^,^ ^11^ ^,^ ^15^.

Eden et al. reported a multinational study of DCD liver transplants, where countries that had available *in situ* and *ex situ* machine perfusions had increased and better DCD utilization rates^17^, demonstrating that NMP can also improve the number of liver transplants performed.

### Costs related to the perfusion machine

The use of *ex situ* perfusion machines represents an exciting new technology that can expand graft usage and reduce waitlist time. However, it is important to analyze how much it would increase the total cost of the transplant.

Raigani et al. conducted the first cost-analysis of end-ischemic NMP, with a median cost of US$ 15,454 to perform NMP in a liver transplant, considering the direct (perfusion device disposables, perfusate components, and point-of-care equipment) and indirect costs (personnel and facility fees, and depreciation of the perfusion device). When compared to the estimated monthly Medicare expenses for the patient with a Model for End-Stage Liver Disease (MELD) of 30, the use of NMP can be justified since it enhances the viability rate usage of the graft^49^.

The first RCT on liver transplantation with NMP (with OrganOx^®^ Metra, OrganOx^®^ Limited, Oxford, United Kingdom) conducted in the UK^31^ ^,^ ^38^ provided the data to elaborate a de novo decision-analytic model that estimates the costs and outcomes of each strategy over a lifetime horizon. The use of NMP was more costly and more effective than SCS preserved organs, representing an important landmark in the NMP cost-effectiveness research.

A Canadian group recently published a cost-effectiveness study comparing NMP (with OrganOx^®^ Metra, OrganOx^®^ Limited, Oxford, United Kingdom) with SCS in liver transplants. They concluded that NMP leads to greater incremental number of well-being-adjusted life years over five years. In addition, NMP was associated with more lives saved and decreased waitlist figures and mortality rates^59^.

One of the advantages of NMP is the possibility to transplant livers that would be discarded under SCS, such as steatotic grafts from older DCD donors and with prolonged warm ischemia time. The number of discarded livers in the United States has been relatively constant since 2005, with a median of 705 grafts discarded annually^44^. According to Raigani et al., if all discarded grafts underwent NMP, an estimated 398 additional livers could meet viability criteria for potential transplantation^49^.

Another major benefit is that machine perfusion can facilitate a lower MELD at transplantation, which has also been recently demonstrated in recent studies^23^ ^,^ ^59^.

Cost-analysis studies on the impact of NMP in liver transplants are still underway. There is a growing demand for more data to evaluate the true impact of this technology on the healthcare system.

### Normothermic regional perfusion

DCD procedures were the standard method for organ procurement for human transplantation in the US prior to the establishment of the Harvard criteria for brain death^19^.

With the increasing shortage of organs and the need for new strategies to enhance organ donation, DCD donors have become an essential resource in the transplant field, thanks to decades of protocol improvements. Early reports on DCD graft outcomes indicated high rates of biliary complications and lower graft survival, which led some centers to hesitate to initially utilize this strategy^13^. However, the development of *in situ* normothermic regional perfusion (NRP) prompted excellent results compared to SCS in reducing the incidence of IC and EAD and improving graft survival in DCD donors^13^. Therefore, DCD became a viable option, and the International Report on Organ Donation and Transplantation Activities in 2023 from the Global Observatory on Donation and Transplantation reported that 25% of all deceased donors were DCD^24^.

NRP consists of a technique that involves rapid cannulation of blood vessels after death is declared, followed by perfusion of the organs that will be used for transplantation. NRP uses *in situ* perfusion with oxygenated blood using an extracorporeal membrane oxygenation circuit or cardiopulmonary bypass circuit through aortic and inferior vena cava cannulas, facilitated by vascular access premortem, or with cannulation being done entirely postmortem, allowing *in situ* viability testing of potential thoracic and abdominal allografts for transplantation^37^ ^,^ ^39^ ^,^ ^61^.

There are three types of techniques: abdominal cannulation (A-NRP), femoral cannulation (femoral A-NRP), and thoracoabdominal cannulation (TA-NRP). The difference between those techniques is which main vessel is cannulated and which organs are being perfused (TA-NRP perfuses organs in the thorax/abdomen, and A-NRP perfuses only abdominal organs).

All techniques perfuse only the organs intended for transplantation and include measures, such as vascular clamps, division of vessels, occlusive vascular balloons, and placement of arterial monitoring lines in non-perfused areas^48^, to prevent meaningful flow to the brain, avoiding any possibility of neuronal perfusion or recovery.

The central legal and ethical question that TA-NRP highlights is whether the perfusion of organs, including the heart, which restarts, negates the circulatory determination of death, which has raised discussions within the transplant community^40^.

### Machine perfusion: the Brazilian perspective

The development of a machine perfusion program in Brazil may face unique challenges. These challenges include limited access to the healthcare system for high-risk liver transplant candidates, long waiting lists, inadequate care for organ donors, reliance solely on DBD donors, since the use of DCD is not ethically accepted, and geographical barriers.

In 2023, Brazil performed approximately 2,365 liver transplants, making it one of the largest transplant countries in the world. However, at the end of that year, there were still 1,391 active patients on the waitlist, with a mortality rate of 19.6% on the list^5^.

Boteon et al. were the first to describe an initial experience of machine perfusion in Brazil. They conducted back-to-base end-ischemic HOPE on six ECD DBD grafts, based on the concept of donororgan matching. Although it was a small population due to the setting of limited resources and complex transplant logistics, the study described a successful introduction of the HOPE procedure, representing a landmark in the Brazilian transplant community^4^.

## CONCLUSIONS

The advent of the perfusion era in liver transplantation rapidly modified the landscape for the foreseeable future. New research and randomized controlled trials are underway to determine whether machine perfusion is a viable and superior alternative to SCS. It remains uncertain how costs will affect healthcare expenditures related to transplants, as machine perfusion has shown improved graft utilization and better patient outcomes. Further studies are necessary to fully understand the benefits and role of perfusion in the transplant process. In developing countries, there are still barriers to overcome, as well as geographical, financial, logistical, and other challenges. Comprehensive and standardized transplant research studies are essential to thoroughly investigate these issues and provide robust data to support the use of machine perfusion.

## Data Availability

The datasets generated and/or analyzed during the current study are available from the corresponding author upon reasonable request.
